# Interleukin-2/interferon-*α*2a/13-retinoic acid-based chemoimmunotherapy in advanced renal cell carcinoma: results of a prospectively randomised trial of the German Cooperative Renal Carcinoma Chemoimmunotherapy Group (DGCIN)

**DOI:** 10.1038/sj.bjc.6603271

**Published:** 2006-08-08

**Authors:** J Atzpodien, H Kirchner, U Rebmann, M Soder, U Gertenbach, M Siebels, J Roigas, R Raschke, S Salm, B Schwindl, S C Müller, S Hauser, C Leiber, E Huland, H Heinzer, S Siemer, B Metzner, H Heynemann, P Fornara, M Reitz, DGCIN – German Cooperative Renal Carcinoma Chemoimmunotherapy Trials Group

**Affiliations:** 1 Fachklinik Hornheide an der Universität Münster, Internistische Onkologie, Dorbaumstr. 300, Münster 48157, Germany; 2 Klinikum Hannover Siloah, Hämatologie/Onkologie, Roesebeckstr. 15, Hannover 30449, Germany; 3 Krankenhaus der Anhaltischen Diakonissenanstalten, Klinik für Urologie, Gropiusallee 3, Dessau 06846, Germany; 4 Universitätsklinikum Heidelberg, Urologische Klinik, Im Neuenheimer Feld 110, Heidelberg 69120, Germany; 5 Allgemeines Krankenhaus der Stadt Hagen, Urologische Klinik, Grünstr. 35, Hagen 58095, Germany; 6 Ludwig-Maximilians-Universität München, Urologische Klinik, Marchioninistr. 15, München 81377, Germany; 7 Charité Berlin, Urologische Klinik, Schuhmannstr. 20-21, Berlin 10098, Germany; 8 Klinikum Ernst-von-Bergmann, Urologische Klinik, Charlottenstr. 72, Potsdam 14467, Germany; 9 Krankenhaus der Barmherzigen Brüder, Urologische Abteilung, Nordallee 1, Trier 54292, Germany; 10 Klinikum Weiden, Urologische Klinik, Söllnerstr. 16, Weiden 92637, Germany; 11 Medizinische Einrichtungen der Universität Bonn, Urologische Klinik, Sigmund-Freud Str. 25, Bonn 53105, Germany; 12 Universitätsklinikum Freiburg, Urologische Abteilung, Hugstetter Str. 55, Freiburg 79106, Germany; 13 Universitätsklinikum Eppendorf, Urologische Klinik, Martinistr. 52, Hamburg 20246, Germany; 14 Universitätsklinik Homburg, Urologische Klinik, Oskar-Orth-Str., Homburg/Saar 66421, Germany; 15 Städtische Kliniken Oldenburg, Hämatologie/Onkologie, Dr.-Eden-Str. 10, Oldenburg 26113, Germany; 16 Universitätsklinikum der, Martin-Luther-Universität Halle-Wittenberg, Urologische Klinik, Ernst-Grube-Str. 40, Halle/Saale 06120, Germany; 17 Europäisches Institut für Tumor Immunologie und Prävention, Bonn 53175, Germany

**Keywords:** metastatic renal cancer, interleukin-2, interferon-*α*, retinoids, chemotherapy

## Abstract

We performed a prospectively randomised clinical trial to compare the efficacy of four subcutaneous interleukin-2-(sc-IL-2) and sc interferon-*α*2a (sc-IFN-*α*2a)-based outpatient regimens in 379 patients with progressive metastatic renal cell carcinoma. Patients with lung metastases, an erythrocyte sedimentation rate ⩽70 mm h^−1^ and neutrophil counts ⩽6000 *μ*l^−1^ (group I) were randomised to arm A: sc-IL-2, sc-IFN-*α*2a, peroral 13-*cis*-retinoic acid (po-13cRA) (*n*=78), or arm B: arm A plus inhaled-IL-2 (*n*=65). All others (group II) were randomised to arm C: arm A plus intravenous 5-fluorouracil (iv-5-FU) (*n*=116), or arm D: arm A plus po-Capecitabine (*n*=120). Median overall survival (OS) was 22 months (arm A; 3-year OS: 29.7%) and 18 months (arm B; 3-year OS: 29.2%) in group I, and 18 months (arm C; 3-year OS: 25.7%) and 16 months (arm D; 3-year OS: 32.6%) in group II. There were no statistically significant differences in OS, progression-free survival, and objective response between arms A and B, and between arms C and D, respectively. Given the known therapeutic efficacy of sc-IL-2/sc-INF-*α*2a/po-13cRA-based outpatient chemoimmunotherapies, our results did not establish survival advantages in favour of po-Capecitabine *vs* iv-5-FU, and in favour of short-term inhaled-IL-2 in patients with advanced renal cell carcinoma receiving systemic cytokines.

The prognosis of metastatic renal carcinoma remains poor. Although this tumour is highly resistant to chemotherapy and hormone therapy, promising results have been reported with the use of molecular agents that is, recombinant cytokines, notably recombinant interleukin-2 (IL-2) and interferon-*α* (IFN-*α*), given intravenously, subcutanously alone or in combination in outpatient regimens with objective response rates between 6 and 31% (Rosenberg *et al*, 1987; Atzpodien *et al*, 1990; Sleijfer *et al*, 1992; Jayson *et al*, 1998; Tourani *et al*, 2003).

When the present trial was planned, various studies focused on the combination of immunmodulator substances and chemotherapeutic agents to increase antitumour activity. In preliminary reports on oral 13-*cis*-retinoic acid (po-13cRA), a cell differentiation regulator, po-13cRA could enhance antitumour efficacy in IL-2/IFN-*α-* or chemoimmunotherapy-treated metastatic renal cell carcinoma patients, with objective response rates between 17 and 42% (Atzpodien *et al*, 1995; Stadler *et al*, 1998). In the presence of pulmonary metastases, locoregional administration of inhaled-IL-2 was reported to yield low toxicity combined with objective response rates of pulmonary disease of 2.5–21% (Lorenz *et al*, 1996; Merimsky *et al*, 2004). Other reports showed that the combination of cytokines with intravenous 5-fluorouracil (iv-5-FU) could increase objective response rates to between 12 and 39% (van Herpen *et al*, 2000; Atzpodien *et al*, 2001, 2004). Preliminary results of a phase II study combining IL-2/IFN-*α* with oral Capecitabine, which is converted to 5-FU *in vivo*, reported objective response rates of 34% (Oevermann *et al*, 2000).

Here, we prospectively compared the long-term therapeutic efficacy of four outpatient combination regimens: arm A (sc-IL-2, sc-IFN-*α*2a, po-13cRA) and arm B (arm A plus inhaled-IL-2) in patients with pulmonary disease, and arm C (arm A plus iv-5-FU) and arm D (arm A plus po-Capecitabine) in all others.

## PATIENTS AND METHODS

### Patients

**Figure 1:**
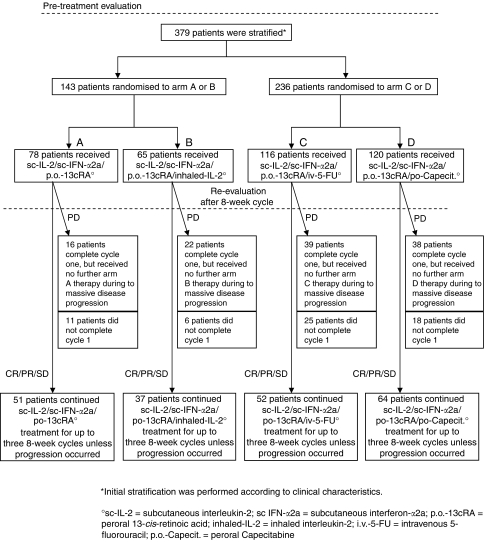
Trial profile.

Three hundred and seventy-nine patients with metastastic renal cell carcinoma were stratified into two groups (Figure 1). Group I patients (*n*=143) were subsequently randomised to arm A (sc-IL-2, sc-IFN-*α*2a, po-13-cRA) or arm B (arm A plus inhaled-IL-2), whereas group II patients (all others; *n*=236) were randomised to arm C (arm A plus iv-5-FU) or arm D (arm A plus po-Capecitabine). Median follow-up of these patients was 18 months (range 0–83 months). Patient pretreatment included radical tumour nephrectomy (*n*=343), radiotherapy (*n*=51), chemotherapy (*n*=11), immunotherapy (*n*=18), chemoimmunotherapy (*n*=19), naturopathic therapy (*n*=2), and others (*n*=7) (Table 1).

Criteria for entry into the trial were as follows: histologically confirmed progressive and irresectable metastatic renal cell carcinoma; an expected survival duration of more than 3 months; Karnofsky performance status >80%; age between 18 and 80 years; white blood cell count >3500 *μ*l^−1^; platelet count >100 000 *μ*l^−1^; haematocrit >30%; serum bilirubin, and creatinine <1.25 of the upper normal limit; no evidence of congestive heart failure, no severe coronary artery disease, no cardiac arrhythmias, no clinically symptomatic CNS disease or seizure disorders, no human immunodeficiency virus infection, no evidence of chronic active hepatitis, no concomitant corticosteroid therapy. In all patients treated, no chemotherapy or immunomodulatory treatment had been performed during the previous 4 weeks. Also, pregnant and lactating women were excluded.

Treatment was approved by the institutional review board, written informed consent was obtained from all patients before entry into the trial. Fifty-four participating centres entered a total of 379 eligible patients into this trial.

### Treatment design

Patients were stratified into two groups according to the clinical characteristics adapted from Lopez-Hänninen *et al* (1996). Group I consisted of patients with lung metastases, an erythrocyte sedimentation rate ⩽70 mm h^−1^, and neutrophil counts ⩽6000 *μ*l^−1^; group II included all other patients. As all treatment regimens were designed to be administered in the outpatient setting, this required selection of patients with good or fair performance status. Upon written receipt of patient pre-treatment evaluation, per centre block randomisation was performed to rule out centre-related statistical bias. Patients stratified to group I or II were randomised according to a per centre 1 : 1 randomisation. Group I patients received arm A (sc-IL2, sc-IFN-*α*2a, po-13-cRA) or arm B (arm A plus inhaled-IL-2), whereas group II patients received either arm C (arm A plus iv-5-FU) or arm D (arm A plus po-Capecitabine).

### Regimens

Treatment arm A consisted of sc-IFN-*α*2a (Roferon, Hoffmann-La Roche, Grenzach-Wyhlen, Germany) (5 × 10^6^ IU m^−2^, day 1, weeks 1+4; days 1, 3, 5, weeks 2–3; 10 × 10^6^ IU m^−2^, days 1, 3, 5, weeks 5–8), sc-IL-2 (Proleukin, Chiron, Emeryville) (10 × 10^6^ IU m^−2^, twice daily, days 3–5, weeks 1+4; 5 × 10^6^ IU m^−2^, days 1, 3, 5, weeks 2+3), and po-13cRA (20 mg 3 × daily) over 8 weeks. Treatment arm B consisted of treatment arm A combined with inhaled-IL-2 (Proleukin, Chiron, Emeryville) (9 × 10^6^ IU/2.5 ml basic solution, four times a day, days 1–5, weeks 2+3 and weeks 5–8); the IL-2 (2 × 18 × 10^6^ IU) was dissolved in 10 ml 5% glucose solution, out of which 2.5 ml (9 × 10^6^ IU IL-2 in solution) was taken for each of the four daily administrations; IL-2 was inhaled using a Salvia Lifetec Jetair inhalator (Kronberg, Germany) constantly providing 3 *μ*m particles. Treatment arm C consisted of arm A plus iv-5-FU (1000 mg m^−2^, day 1, weeks 5–8). Treatment arm D consisted of treatment arm A combined with po-Capecitabine (1000 mg m^−2^ twice daily, days 1–5, weeks 5–8).

Eight-week treatment cycles were repeated for up to three courses unless progression of disease occurred. Patients with an unchanged tumour response in cycle two did not receive a subsequent third treatment cycle.

Re-evaluation of the patients tumour status was performed between treatment cycles. Arm A, B, C, and D patients received a mean of 1.6, 1.6, 1.5, and 1.6 8-week cycles, respectively (range 1–6). Concomitant medication was given as needed to control adverse effects of chemoimmunotherapy. Sixty patients (15.8%) (arm A: 14%; arm B: 9.2%; arm C: 21.6%; arm D: 15%) did not complete cycle one owing to early disease progression before the first evaluation (2.9%), intolerance (8.7%), death during therapy (1.6%), patients’ wish (2.1%), and non-compliance (0.5%), respectively. Less than 4% of patients required in-patient care throughout treatment. All patients were seen at regular weekly or bi-weekly intervals by oncologic specialists; additional care was provided whenever needed.

### Assessment of response, survival, and toxicity

Response to therapy was evaluated according to World Health Organization (WHO) criteria on an intent-to-treat basis. All responses were reviewed by Board-certified expert radiologists. In case of progression upon first re-evaluation after 8 weeks of treatment, progression-free survival (PFS) was calculated at 0 months. Survival was measured from start of therapy to date of death or to the last known date to be alive. All patients had to be followed up for survival for at least 3 years as cutoff.

Systemic maximum toxicity was evaluated using a grading system adapted from the WHO.

### Statistical analysis

The statistical end points in our analysis were (1) OS, (2) PFS, and (3) objective response of patients. The probability of OS and PFS was plotted over time according to the method of Kaplan and Meier (1958). Statistical significance was assessed using the log-rank test.

The 3-year survival rates were hypothesised to show a 20% advantage of arm B over arm A (40 *vs* 20%), and a 15% advantage of arm D over arm C (30 *vs* 15%). Using an *α* of 0.05 (one-sided), a sample size of 64 patients each (arm A and arm B) and 95 patients each (arm C and arm D) was needed to have 80% power to statistically establish the assumed difference in 3-year survival rates. To meet these statistical end points, randomisation was performed 1 : 1 for groups I and II, respectively.

## RESULTS

A total of 379 metastatic renal carcinoma patients were treated: 143 group I patients with pulmonary metastases (median OS: 21 months) were randomised to receive sc-IL-2, sc-IFN-*α*2a, and po-13cRA (arm A); or arm A plus inhaled-IL-2 (arm B); 236 group II patients (median OS: 17 months) were randomised to receive arm A plus iv-5-FU (arm C); or arm A plus po-Capecitabine (arm D).

### Treatment response

Eight arm A (sc-IL-2/sc-IFN-*α*2a/po-13cRA) treated patients (10%) achieved a complete response and 15 patients (19%) had a partial remission (Table 2). The overall objective response rate was 29% (95% CI 19, 40). Twenty-eight patients (36%) showed disease stabilisation and 27 patients (35%) exhibited continuous disease progression despite therapy.

In arm B (arm A plus inhaled-IL-2), there were eight complete responders (12%) and 12 partial responders (19%), with an overall objective response rate of 31% (95% CI 20, 44). Seventeen patients (26%) had disease stabilisation, and in 28 patients (43%) a continuous disease progression was observed.

In arm C (arm A plus iv-5-FU), four patients (3%) achieved a complete response and 18 patients (16%) had a partial remission. The overall objective response rate was 19% (95% CI 13, 27). Thirty patients (26%) showed disease stabilisation and 64 patients (55%) exhibited continuous disease progression despite therapy.

Nine arm D (arm A plus po-Capecitabine) treated patients (7%) achieved a complete response and 23 patients (19%) had a partial remission. The overall objective response rate was 26% (95% CI 19, 35). Thirty-two patients (27%) showed disease stabilisation, and in 56 patients (47%) a continuous disease progression was observed.

### Progression-free survival

**Figure 2:**
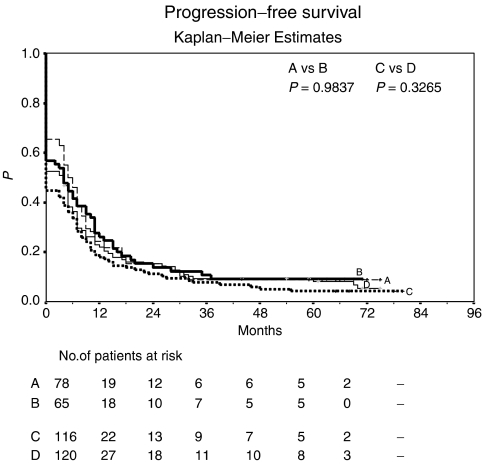
Progression-free survival: Kaplan–Meier estimates. Arm A, sc-IL-2, sc-IFN-*α*2a, po-13-*cis*-retinoic acid; *n*=78; median PFS, 5 months; 3-year PFS, 8.8%. Arm B, sc-IL-2, sc-IFN-*α*2a, po-13-*cis*-retinoic acid, inhaled-IL-2; *n*=65; median PFS, 4 months; 3-year PFS, 10.8%. Arm C, sc-IL-2, sc-IFN-*α*2a, po-13-*cis*-retinoic acid, iv-5-FU; *n*=116; median PFS, 0 months; 3-year PFS, 7.8%. Arm D, sc-IL-2, sc-IFN-*α*2a, po-13-*cis*-retinoic acid, po-Capecitabine; *n*=120; median PFS, 4 months; 3-year PFS, 9.3%.

Seven patients (9%) in arm A (sc-IL-2/IFN-*α*2a/po-13cRA), six patients (9%) in arm B (arm A plus inhaled-IL-2), five patients (4%) in arm C (arm A plus iv-5-FU), and nine patients (8%) in arm D (arm A plus po-Capecitabine) remained progression-free at last follow-up. Patients reached a median PFS of 5 months in arm A (3-year PFS: 8.8%) and 4 months in arm B (3-year PFS: 10.8%). Median PFS was 0 months in arm C (3-year PFS: 7.8%) and 4 months in arm D (3-year PFS: 9.3%) (Figure 2). There was no statistically significant difference in PFS between arms A and B (*P*=0.9837), and between arms C and D (*P*=0.3265).

### Overall survival

**Figure 3:**
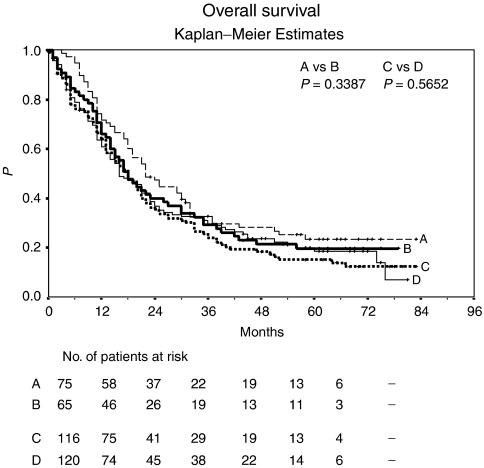
Overall survival: Kaplan–Meier estimates. Arm A, sc-IL-2, sc-IFN-*α*2a, po-13-*cis*-retinoic acid; *n*=78; median OS, 22 months; 3-year OS, 29.7%. Arm B, sc-IL-2, sc-IFN-*α*2a, po-13-*cis*-retinoic acid, inhaled-IL-2; *n*=65; median OS, 18 months; 3-year OS, 29.2%. Arm C, sc-IL-2, sc-IFN-*α*2a, po-13-*cis*-retinoic acid, iv-5-FU; *n*=116; median OS, 18 months; 3-year OS, 25.7%. Arm D, sc-IL-2, sc-IFN-*α*2a, po-13-*cis*-retinoic acid, po-Capecitabine; *n*=120; median OS, 16 months; 3-year OS, 32.6%.

Seventeen patients (22%) in arm A (sc-IL-2/sc-IFN-*α*2a/po-13cRA), 13 patients (20%) in arm B (arm A plus inhaled-IL-2), 16 patients (14%) in arm C (arm A plus iv-5-FU), and 22 patients (18%) in arm D (arm A plus po-Capecitabine) continued to be alive at last follow-up. Median OS was 22 months in arm A (3-year OS: 29.7%), 18 months in arm B (3-year OS: 29.2%), 18 months in arm C (3-year OS: 25.7%), and 16 months in arm D (3-year OS: 32.6%) (Figure 3). There was no statistically significant difference in OS between arms A and B (*P*=0.3387), and between arms C and D (*P*=0.5652).

### Treatment toxicity

All four sc-IL-2/sc-IFN-*α*2a/po-13-cRA-based therapies were moderately tolerated and could be administered in the outpatient setting. Most side effects were limited to WHO grades I and II, and no toxic deaths occurred. All toxicities reversed spontaneously following completion of chemoimmunotherapy.

Table 3 summarises all grade I/II and III/IV treatment-related adverse effects. More than 5% of patients experienced grade III or IV treatment-related anorexia (24% arm A, 26% arm B, 18% arm C, 26% arm D), malaise (19% arm A, 8% arm B, 11% arm C, 28% arm D), nausea/vomiting (10% arm B), chills (9% arm C, 7% arm D), fever (9% arm D), and respiratory distress (7% arm A, 7% arm C).

A total of 5% of arm A (sc-IL-2/IFN-*α*2a/po-13-cRA), 8% of arm B (arm A plus inhaled-IL-2), 12% of arm C (arm A plus iv-5-FU), and 8% of arm D (arm A plus po-Capecitabine) patients discontinued treatment owing to toxicity.

## DISCUSSION

In this prospectively randomised trial, we reported the results of 379 patients with progressive metastatic renal cell carcinoma who received (A) sc-IL-2, sc-IFN-*α*2a, po-13cRA, (B) arm A plus inhaled-IL-2, (C) arm A plus iv-5-FU, or (D) arm A plus po-Capecitabine.

We showed that patients with lung metastases, an erythrocyte sedimentation rate ⩽70 mm h^−1^, and neutrophil counts ⩽6000 *μ*l^−1^ (group I) achieved a median OS of 22 months (arm A) and 18 months (arm B), whereas all other patients (group II) reached a median OS of 18 months (arm C) and 16 months (arm D), with no statistically significant differences. Overall, other authors reported a median OS of 17 months (sc-IL-2/sc-IFN-*α*/po13-cRA) (Stadler *et al*, 1998), 19 months (sc-IL-2/sc-IFN-*α*/inhaled-IL-2) (Huland *et al*, 1994), and 11–27 months (sc-IL-2/sc-IFN-*α*/po-13cRA/iv-5-FU) (Soori *et al*, 2002; Atzpodien *et al*, 2004).

It should be noted that the primary end point of the current trial was not reached, given 3-year OS-rates of 29.7% (arm A) and 29.2% (arm B) in group I, and 25.7% (arm C) and 32.6% (arm D) in group II, with no statistically significant differences.

There were also no significant differences in PFS and objective response between arms A (sc-IL-2/sc-IFN-*α*/po-13cRA) and B (arm A plus inhaled-IL-2), and between arms C (arm A plus iv-5-FU) and D (arm A plus po-Capecitabine). Inhaled IL-2 in poor-risk patients unfit for systemic cytokine therapy has been reported to induce remarkable effects on lung metastases leading to objective responses between 2.5–21% (inhaled-IL-2) (Lorenz *et al*, 1996; Merimsky *et al*, 2004) and 47% (inhaled-IL-2/10% sc-IL-2/INFa) (Huland *et al*, 1994). In this multicentre trial, inhaled-IL-2 (arm B) at a dose 5.3-fold the subcutaneous (sc) dose did not significantly enhance treatment efficacy when compared to arm A. However, there are major differences compared with other reports using inhaled-IL-2 in patient selection, complementary therapy, dose distribution, and treatment time. In this trial, we treated patients with good performance status in addition to an effective systemic chemoimmunotherapy, where further improvement of objective responses may be unlikely. We used inhalation for a mean of 1.6 cycles or 64 inhalation days compared to mean treatment times of 260 inhalation days (301 days in total) reported in poor-risk patients earlier (Huland *et al*, 2003). Our results confirmed safety and tolerance of inhaled-IL-2 added to effective chemoimmunotherapy.

Although the use of po-Capecitabine (arm D), as 5-FU pro-drug, yielded objective response rates of 34% in preliminary studies (Oevermann *et al*, 2000), here, it did not significantly impact on objective response rates and survival when compared to arm C (sc-IL-2/sc-IFN-*α*2a/po-13cRA/iv-5-FU). However, with comparable safety and tolerance, the oral application of Capecitabine presented a potential treatment improvement in the palliative setting.

In the present trial, all four treatment arms were moderately tolerated; yet, more than 5% of patients experienced grade III or IV treatment-related side effects, notably, anorexia and malaise. It was previously suggested that grade III and IV toxicities and reduced quality of life may be more prevalent in a po-13cRA-based treatment (Motzer *et al*, 2000; Atzpodien *et al*, 2004; Aass *et al*, 2005). Here, the observed antitumour effects of po-13cRA must be weighed against the toxicity profile in patients with metastatic renal cell carcinoma.

In summary, given the known therapeutic efficacy of sc-IL-2/sc-INF-*α*2a/po-13cRA-based outpatient chemoimmunotherapies, our results did not establish survival advantages in favour of po-Capecitabine *vs* iv-5-FU, and in favour of short-term inhaled-IL-2 in patients with advanced renal cell carcinoma receiving systemic cytokines.

## Figures and Tables

**Table 1:** Patients characteristics and pretreatment

	**Therapy^a^**				
**Characteristic**	**Arm A**	**Arm B**	**Arm C**	**Arm D**	**All patients**
Entered	78	65	116	120	379
					
*Age (years)*					
Median	61	60	60	60	60
Range	28–79	42–75	32–78	35–75	28–79
					
*Sex*					
Male	52	48	82	93	275
Female	26	17	34	27	104
					
*Pretreatment*					
Radical tumour nephrectomy	70	57	107	109	343
Radiotherapy	7	6	19	19	51
Chemotherapy	2	2	3	4	11
Immunotherapy	2	3	4	9	18
Chemoimmunotherapy	3	4	4	8	19
Naturopathic	0	0	0	2	2
Others	1	1	5	0	7
					
*Metastatic sites*					
Lung/pleural	78	65	57	70	270
Lymph nodes	28	13	42	46	129
Bone	8	12	28	32	80
Liver	6	6	29	28	69
Contralateral kidney	5	3	10	8	26
Adrenals	3	2	5	7	17
Soft tissue	2	0	10	11	23
CNS	0	0	4	1	5
Others^b^	9	4	22	12	47

a Arm A (sc-interleukin-2/sc-interferon-*α*2a/po-13-cis-retinoic acid); arm B (sc-interleukin-2/sc-interferon-*α*2a/po-13-cis-retinoic acid/inhaled-IL-2); arm C (sc-interleukin-2/sc-interferon-*α*2a/po-13-cis-retinoic acid/iv-5-fluorouracil); and arm D (sc-interleukin-2/sc-interferon-*α*2a/po-13-cis-retinoic acid/po-Capecitabine).

b Including local relapse, thyroid, spleen, dermal, mammae.

**Table 2:** Response to therapy according to WHO criteria (intent to treat)

		**Response**				
**Therapy**		**Complete response**	**Partial response**	**Stable disease**	**Progressive disease**	**Total**
*Arm A*						
*sc-interleukin-2/sc-interferon-α2a/*	*n*	8	15	28	27	78
*po-13-cis-retinoic acid*	%	10%	19%	36%	35%	100%
Obj. Resp. (95% CI)		29% (19–40%)				
						
*Arm B*						
*sc-interleukin-2/sc-interferon-α2a/*	*n*	8	12	17	28	65
*po-13-cis-retinoic acid/inhaled-interleukin-2*	%	12%	19%	26%	43%	100%
Obj. Resp. (95% CI)		31% (20–44%)				
						
*Arm C*						
*sc-interleukin-2/sc-interferon-α2a/*	*n*	4	18	30	64	116
*po-13-cis-retinoic acid/iv-5-fluorouracil*	%	3%	16%	26%	55%	100%
Obj. Resp. (95% CI)		19% (13–27%)				
						
*Arm D*						
*sc-interleukin-2/sc-interferon-α2/*	*n*	9	23	32	56	120
*po-13-cis-retinoic acid/po-Capecitabine*	%	7%	19%	27%	47%	100%
Obj. Resp. (95% CI)		26% (19–35%)				

**Table 3:** Systemic maximum toxicity

	**% Patients**							
	**Arm A^a^**		**Arm B^a^**		**Arm C^a^**		**Arm D^a^**	
	**I/II**	**III/IV**	**I/II**	**III/IV**	**I/II**	**III/IV**	**I/II**	**III/IV**
Fever^b^	76	2	67	4	77	4	74	9
Chills	69	5	61	4	68	9	67	7
Malaise	60	19	67	8	77	11	59	28
Nausea/vomiting	60	2	52	10	70	5	58	2
Anorexia	48	24	35	26	44	18	44	26
Diarrhoea	33	—	29	5	38	4	38	2
Respiratory distress	41	7	48	4	41	7	39	4
Mucositis	52	2	48	4	46	2	45	—
Hypotension	38	—	19	—	23	2	23	—
Alopecia	3	—	36	—	23	2	28	—
Arrythmias	24	2	10	—	12	4	13	2
CNS/disorientation	26	—	19	—	22	—	26	2
Paresthesias	21	—	5	—	11	—	12	—
Fluid retention/oedema	12	—	14	—	5	2	13	2
Leucocyte counts	10	2	6	3	12	—	6	2
Thrombocyte counts	4	—	—	—	3	—	8	2
Haemoglobin levels	16	—	14	5	31	2	21	5

a Arm A (sc-interleukin-2/sc-interferon-*α*2a/po-13-cis-retinoic acid); arm B (sc-interleukin-2/sc-interferon-*α*2a/po-13-cis-retinoic acid/inhaled-IL-2); arm C (sc-interleukin-2/sc-interferon-*α*2a/po-13-cis-retinoic acid/iv-5-fluorouracil); and arm D (sc-interleukin-2/sc-interferon-*α*2a/po-13-cis-retinoic acid/po-Capecitabine).

b All patients received a standard regimen of fever-reducing treatment employing p.o. paracetamol.
